# Development of Biocompatible Electrospun PHBV-PLLA Polymeric Bilayer Composite Membranes for Skin Tissue Engineering Applications

**DOI:** 10.3390/molecules29092049

**Published:** 2024-04-29

**Authors:** Muddasar Jamal, Faiza Sharif, Muhammad Shozab Mehdi, Muhammad Fakhar-e-Alam, Muhammad Asif, Waleed Mustafa, Mustehsan Bashir, Sikandar Rafiq, Mohamad Azmi Bustam, Saif-ur-Rehman, Kholood A. Dahlous, Mohamed F. Shibl, Noora H. Al-Qahtani

**Affiliations:** 1 Interdisciplinary Research Centre in Biomedical Materials, COMSATS University Islamabad, Lahore Campus, Lahore 54000, Pakistan; muddasar_18002825@utp.edu.my (M.J.); waleed_mustafa@cuilahore.edu.pk (W.M.); saifzciit@gmail.com (S.-u.-R.); 2 Department of Chemical Engineering, COMSATS University Islamabad, Lahore Campus, Lahore 54000, Pakistan; 3 Department of Chemical Engineering, Universiti Teknologi PETRONAS, Bandar Seri Iskandar 32610, Perak, Malaysia; azmibustam@utp.edu.my; 4 Ghulam Ishaq Khan Institute of Engineering Sciences and Technology, Topi 23640, Pakistan; shozab@giki.edu.pk; 5 Department of Physics, Government College University Faisalabad, Allama Iqbal Road, Faisalabad 38000, Pakistan; fakharphy@gmail.com (M.F.-e.-A.);; 6 Department of Plastic, Reconstructive Surgery and Burn Unit, King Edward Medical University, Lahore 54000, Pakistan; mmbashir1@gmail.com; 7 Department of Chemical, Polymer and Composites Materials Engineering, University of Engineering and Technology-Lahore, New Campus, Lahore 39161, Pakistan; sikander@uet.edu.pk; 8 Department of Chemical Engineering, ProcESS-Process Engineering for Sustainable System, KU Leuven, Celestijnenlaan 200F, B-3001 Leuven, Belgium; 9 Department of Chemistry, College of Science, King Saud University, Riyadh 11451, Saudi Arabia; kdahloos@ksu.edu.sa; 10 Chemistry Department, Faculty of Science, Cairo University, Cairo 12613, Egypt; mfshibl@cu.edu.eg; 11 Center for Advanced Materials, Qatar University, Doha P.O. Box 2713, Qatar

**Keywords:** bilayer composites, polymeric membranes, PHBV, PLLA, skin regeneration

## Abstract

Bilayer electrospun fibers aimed to be used for skin tissue engineering applications were fabricated for enhanced cell attachment and proliferation. Different ratios of PHBV-PLLA (70:30, 80:20, and 90:10 *w*/*w*) blends were electrospun on previously formed electrospun PHBV membranes to produce their bilayers. The fabricated electrospun membranes were characterized with FTIR, which conformed to the characteristic peaks assigned for both PHBV and PLLA. The surface morphology was evaluated using SEM analysis that showed random fibers with porous morphology. The fiber diameter and pore size were measured in the range of 0.7 ± 0.1 µm and 1.9 ± 0.2 µm, respectively. The tensile properties of the bilayers were determined using an electrodynamic testing system. Bilayers had higher elongation at break (44.45%) compared to the monolayers (28.41%) and improved ultimate tensile strength (7.940 MPa) compared to the PHBV monolayer (2.450 MPa). In vitro cytotoxicity of each of the scaffolds was determined via culturing MC3T3 (pre-osteoblastic cell line) on the membranes. Proliferation was evaluated using the Alamar Blue assay on days 3, 7, and 14, respectively. SEM images of cells cultured on membranes were taken in addition to bright field imaging to visually show cell attachment. Fluorescent nuclear staining performed with DAPI was imaged with an inverted fluorescent microscope. The fabricated bilayer shows high mechanical strength as well as biocompatibility with good cell proliferation and cell attachment, showing potential for skin substitute applications.

## 1. Introduction

Skin covers the largest surface area of the human body and forms a barrier between the internal and external environment [1,2]. Healthy skin is significant to maintain the normal homeostasis of the human body [3]. The skin has a complex multilayer structure, which is composed of cells, fibers, capillaries, hair follicles, and additional cell networks (ECMs). Skin generally comprises three layers, an upper epidermis, a middle dermis, and the lower hypodermis. The epidermis consists of keratinocytes, the dermis is composed of fibroblasts and collagen, and the third, innermost layer, the hypodermis, possesses lipocytes and collagen [2].

Throughout the lifetime of an individual, the skin constantly heals itself and regains its integrity through a harmonized interaction between keratinocytes, fibroblasts, melanocytes, and endothelial cells in addition to the involvement of certain signaling molecules [4]. The same process is used for the repair of injured or damaged skin tissue. Modern medical interventions help wound healing and injury recovery via tissue grafting with autologous, allogenic, or xenogeneic skin structures [5,6]. However, this process involves donor site morbidity, which can be avoided by replacing autologous tissue with synthetic and natural skin substitutes [7,8]. Skin substitutes are used for the regeneration and wound healing of skin, mainly for burns, and testing of cosmetics and drugs for skin.

According to the World Health Organization (WHO), burns cause 180,000 deaths every year whereby low and middle-income countries are the major victims [9]. Over 1,000,000 people in India and 173,000 in Bangladesh are moderately or severely burnt every year. In Bangladesh, Colombia, Egypt, and Pakistan, 17% of children with burns have a temporary disability and 18% have a permanent disability [10]. Only a small percentage of patients have access to reconstructive procedures, while the rest of them remain untreated and handicapped [11,12].

Many skin healing substitutes are available in the market, each with its limitations. Some of these products act as wound dressings while others are impregnated with cells that are incapable of surviving long enough to heal the wound completely [3,12]. However, imitating natural skin with all its functions has not been achieved effectively so far. To achieve optimum mechanical properties, multilayered structures to cater for appendages and respective cellular populations, achieving innervation and vascularization, and the absence of organogenesis are major deficiencies of current models [13,14]. Other disadvantages of these models are fragility microbial contamination, lower engraftment rates, healing delay, and high cost. This prevents the substitutional skin from being fully functional, like natural skin [15].

Distinctive techniques have been utilized for the preparation of polymer nanofibers in recent years, for example, electrospinning [16,17], phase separation [18], self-assembly [19,20], drawing [21], and template synthesis [22,23]. Electrospun polymer nanofibers are used in various applications, such as biosensors, optical electronics, wound healing, nonwoven fabrics, filtration, drug delivery, and scaffolds for tissue engineering. Because of environmental concerns and sustainability problems, renewable and biocompatible polymers are getting more attention [24,25]. Bio-nanocomposites are a class of nanocomposite materials. Here, the prefix “bio” suggests that inorganic/petroleum-based constituents are replaced with bio-based, environmentally friendly alternatives [26]. In recent research, the commonly used bio-nanocomposites are HA-PLA, HA-PCL, PU-PLA, PAL-PCL, and PHBV-PLLA [27].

In this study, PHBV (Polyhydroxy butyrate-co-hydroxy valerate) was selected for its biocompatible and biodegradable nature. Furthermore, it is easy to be electrospun into nanofibrous membrane layers, resulting in high tensile strength [28]. PLLA (Poly l-lactic acid) was selected as the second polymer, which is also known for its characteristics and biocompatibility [29,30]. PLLA has been widely used for various applications ranging from pelvic floor repair material [31] to the preparation of biodegradable membranes with the combination of different polymers [32].

The novelty of this work lies in the fabrication of a nano-porous/microporous-nano-porous bilayer membrane that has a skin-like bilayer architectural structure. A thin compact layer of PHBV was first formed through electrospinning; then, PHBV-PLLA electrospun fibers were developed on this PHBV monolayer to form a bilayer to enhance cell attachment and proliferation. The prepared bilayer mats were characterized with FTIR, the Electrodynamic Fatigue Testing system, and SEM analysis for structural, mechanical, and morphological properties, respectively. Biocompatibility was assessed by culturing the MC3T3 (pre-osteoblastic) cell line and Alamar Blue assay on days 3, 7, and 14, respectively. To gain more insight, cell attachment was observed through SEM micrograph and fluorescent nuclear staining for investigating an electrospun bilayer of the PHBV/PHBV-PLLA biodegradable polymeric matrix that can be optimized further for skin repair applications.

## 2. Results and Discussion

### 2.1. SEM for Electrospun PHBV-PLLA Bilayer Membranes

**Figure 1:**
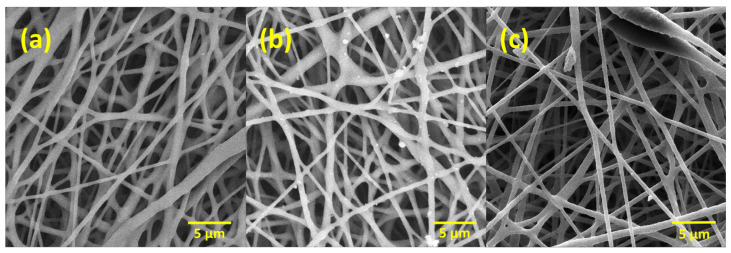
Electrospun bilayer PHBV-PLLA membranes at different magnifications. (**a**) 90:10, (**b**) 80:20, and (**c**) 70:30 (*w*/*w*) ratio.

Figure 1 shows SEM images of PLLA-PHBV bilayer membranes. The surface of these membranes has a randomly uniform fibrous morphology with a 3D appearance [33]. A porous structure and uniform random fiber deposition are the main features observed in all the membranes [34]. Figure 1a–c show the bilayer membranes 90:10, 80:20, and 70:30 *w*/*w* PHBV-PLLA at magnifications of 5k×, respectively. The arrangement and distribution of fibers give a microporous/nano-porous nature to the membranes [35], which are a prerequisite for a biocompatible membrane to mimic the native extracellular matrix of the skin.

**Figure 2:**
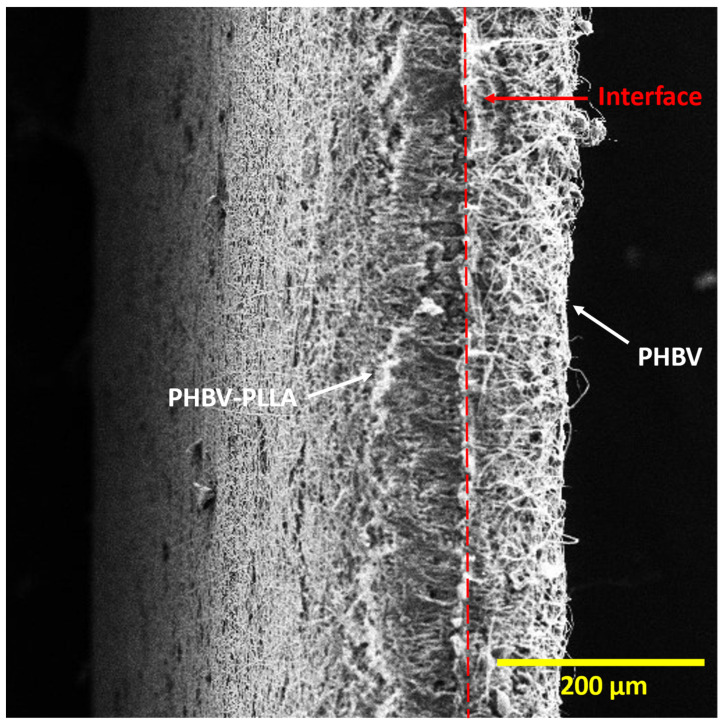
Cross-sectional view of the Bilayer PHBV-PLLA membranes. (White arrows: individual layers, red line: interface between two layers).

Figure 2 shows the cross-sectional images of PHBV-PLLA bilayer membranes, taken at a magnification of 200×. In the bilayer membrane, there is a partition between the PHBV-PLLA blend fibers and the PHBV fiber layer. A dashed red line in the middle of the membrane indicates the interface of the bilayer structure of the membrane having a PHBV layer on the left and a PHBV-PLLA layer on the right (Figure 2). Interlinking fibers show a clear interlayer adherence and hence are a determinant of consistency in mechanical load distribution among the polymeric layers. The porous structure of bilayer membranes helps in cellular migration through the pores and attachment due to the higher available surface area of the fibers, which increases cell proliferation and improves biocompatibility.

### 2.2. Pore Diameter and Fiber Thickness Analysis

**Figure 3:**
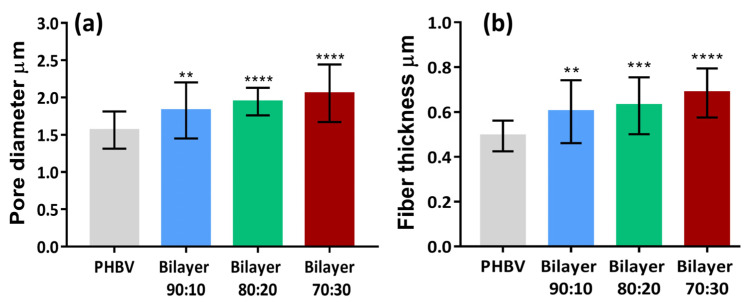
Comparison of (**a**) pore diameter and (**b**) fiber thickness of bilayer PHBV-PLLA membranes with monolayer PHBV. (** *p* < 0.01, *** *p* < 0.001, **** *p* < 0.0001).

Pore diameter and fiber thickness play an important role in cell proliferation. The pore diameter and fiber thickness of the three fabricated bilayer membranes are shown in Figure 3. The pore void of bilayer 90:10 was measured as 1.8 µm, that of 80:20 was 1.9 µm, and 70:30 was 2.1 µm. The pore diameter was higher in membranes containing higher PLLA content. Nano-pored scaffolds with pore voids less than 1 μm can be applied to improve cell–surface interaction, and larger pore size (around 1–3 μm) is needed for cell-to-cell interaction on anchorage-dependent cell populations [36]. Therefore, all three membranes can allow cell-to-cell interactions for tissue engineering applications. Similarly, the fiber thickness of the 90:10 bilayer was 0.60 µm, 0.65 µm for 80:20, and 0.70 µm for 70:30, as shown in Figure 3. The difference between the means of fiber thickness of each type of bilayer is significant compared to the control. SEM analysis shows that the fibers and pore diameters were in the range of µm and provide good anchorage points for cells to the membranes with enhanced cell proliferation and migration through these interconnected pores. The fibrous architecture of these electrospun fibers mimics the collagen fibers of the extracellular matrix, providing a large surface area for cell attachment and an open porous structure for cells to migrate into the scaffolds, which is essential for good tissue integration [35], as discussed earlier.

### 2.3. Porosity Measurement

The porosity of the membranes and the apparent densities were calculated using the formulas stated in the materials and methods section and graphically represented in Figure 3. Membrane porosity ranging from 60 to 90% is preferred for the penetration of the cells [37]. For cellular integration, electrospinning creates pores of appropriate size by dispensing fibers at random orientations. The porosity of all the membranes is within the range of 63–70%, as given in Table 1. This indicates that the porosity values reached with electrospinning for this polymeric combination are acceptable for a given thickness appropriate for the epidermis [38] and possibly extendable to the dermis with more fibrous deposition.

### 2.4. FTIR Analysis

**Figure 4:**
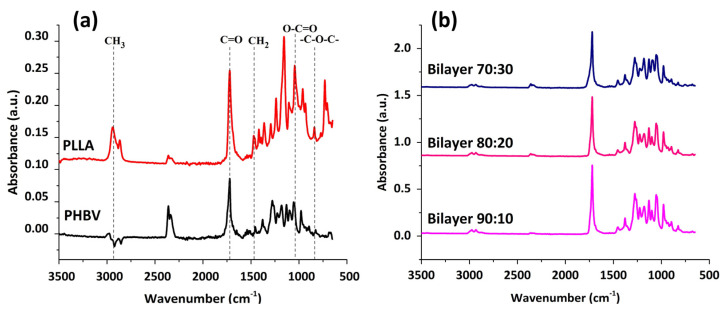
(**a**) FTIR for PHBV and PLLA membranes with characteristic peaks. (**b**) FTIR spectral comparison between different formulations of bilayer PHBV-PLLA membranes.

Figure 4 shows the FTIR spectra of PHBV and PLLA membranes and stacked spectra of bilayers having PHBV-PLLA electrospun membranes with three blend compositions. The FTIR spectra of PHBV show a strong band of around 1720 accounting for C=O stretching. Symmetric -C-O-C bonding is exhibited in the range of 800–975 cm^−1^,whereas asymmetric stretching for -C-O-C signals is seen between 1060 and 1150 cm^−1^. In spectra obtained from PLLA, CH_3_ stretching is observed in a range of 3000–2950 cm^−1^, whereas C=O stretching is similar to that in the PHBV band. CH_2_ and CH_3_ bending were found at 1452 and 1279 cm^−1^, respectively. The O-C=O stretching around 1200–1090 cm^−1^ exhibits the characteristic ester groups. The IR spectra are a combination of the IR peaks from PLLA and PHBV and were found to be 3000, 2925, and 2845 cm^−1^ for CH, CH_2_, and CH_3_, respectively. Other peaks include 1720 cm^−1^ for C=O stretching, 1440 and 1370 cm^−1^ for CH_2_ and CH_3_ for bending, and 1270–1050 cm^−1^ for C-O-C stretching. 975 cm^−1^ was found for CH_2_ in-plane bending of PHBV and 869 cm^−1^ for O-C-H-CH_3_, which are similar to the trend reported in HC Chang et al. [39]. As is evident from the graphs, there are no considerable peak shifts for the bilayers with compositional variations. By changing the compositions, fiber morphology and the porous structure of the membranes are changing, but no chemical change occurred, which resulted in no noticeable peak shifts. Considerable peak intensities are observed in comparison to other specimens for the bilayer samples owing to the better reflectance and absorbance by the samples due to the formation of the bilayer [40].

### 2.5. Mechanical Testing

**Figure 5:**
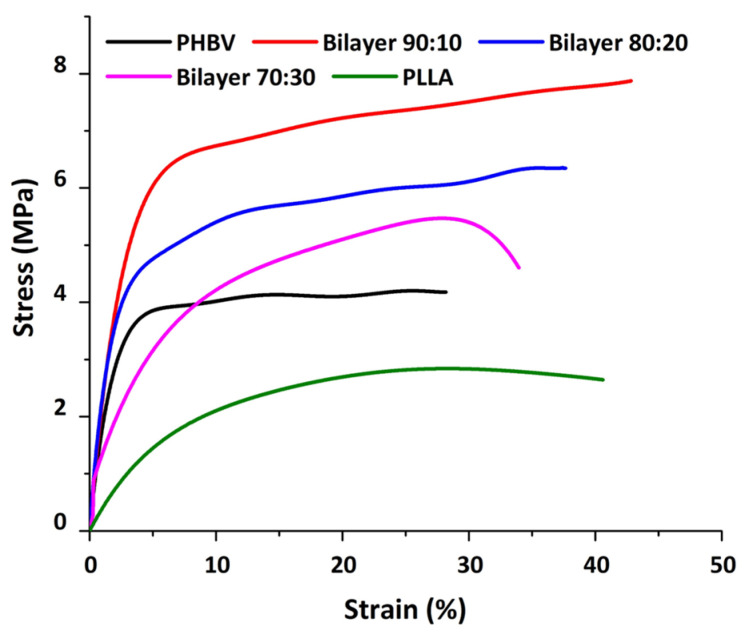
Stress and strain graph showing the mechanical behavior of membranes.

Mechanical testing was conducted on electrospun membranes to determine the tensile strength and Young’s modulus of the membranes. Figure 5 shows the stress and strain curves for all the membranes. These membranes have an overall ductile behavior under applied stress. The curves show a proportional increase in the stress and strain ratio at the beginning, where Hooke’s law is being followed. All the membranes have a strain percentage in this linear portion under less than 10% [28].

The stress–strain curve of the PHBV membrane in comparison to the bilayer membrane suggests the increase in the stress/strain of bilayers with the addition of PLLA to the PHBV polymer. All bilayers have higher mechanical strength as compared to the PHBV monolayer. Overall, the mechanical strength decreased from the composition of 90:10 to 80:20 and then 70:30 *w*/*w*, respectively. This can be attributed to the PLLA ratio in the blend; the higher the ratio, the lower the mechanical strength (Table 2), as PLLA is known to have lower mechanical strength as compared to the PHBV polymer [17]. However, the overall tensile strength was better in the bilayer compared to PHBV alone, which may be due to the PHBV bottom layer, which provides support to the blended layer and enhances the mechanical properties of the bilayer membranes.

As the amount of PLLA increases from 10% to 30%, the diameter of the fiber increases, as evident from Figure 5. Hence, it is presumed that the number of fibers per unit area of the electrospun membrane decreases, resulting in a decrease in the mechanical strength. This is also apparent from the fiber thickness and void size, which increased with the addition of PLLA in the blend. It is important to note that at higher thicknesses, comparable to natural human skin, both monolayers and bilayer membranes can show much higher modulus values with improved elongation at break (Table 2); in addition, different regions of skin yield different Young’s modulus values, as measured by Griffin et al., i.e., 1.28 MPa [41]. Since these values show comparably improved mechanical properties, a bilayer scheme provides evidence as a better option to use as a substitute to mimic the skin’s mechanical behavior.

### 2.6. Cell Culture Evaluation

In vitro cytotoxicity of each of the membranes was determined by culturing MC3T3 (pre-osteoblastic cell line) on the membranes. Cell proliferation was evaluated via Alamar Blue assay on days 3, 7, and 14. SEM images of cells cultured from membranes were taken as well as inverted microscopic imaging.

#### 2.6.1. Alamar Blue Assay

**Figure 6:**
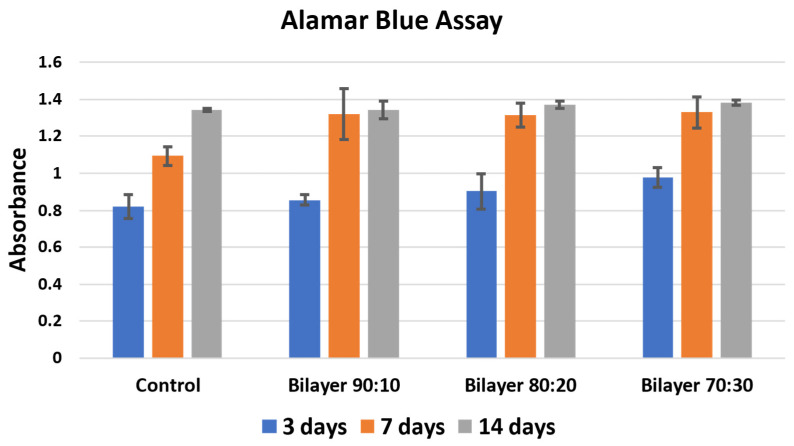
Cell proliferation Alamar Blue assay of bilayer PHBV-PLLA membranes. (Sidak’s multiple comparison test showed no significant values for *p*).

Cell proliferation assay was performed using Alamar Blue stain. The experiment was performed for 3, 7, and 14 days via co-culturing the membranes, and the results were then compared with control. Figure 6 shows the absorbance results of the control, which shows cells seeded in the media without membrane, and bilayer 90:10, 80:20, and 70:30 *w*/*w* membranes for 3, 7, and 14 days, respectively [42,43].

It is evident in Figure 6 that there is an increase in cell proliferation of bilayer PHBV-PLLA membranes compared to the control. The proliferation increased with time, i.e., from 3 to 7 with visible viability increment and then up to 14 days for all the membranes and in the control. The absorbance values after incubation with the Alamar Blue stain are an indicator of proliferation. The higher the absorbance, the higher the biocompatibility and cell proliferation of the tested material [44]. The 70:30 bilayer membrane showed the highest proliferation, which may be due to higher void size for cell integration and/or due to higher participation of biocompatible PLLA. An increase in percentage proliferation as compared to control after 3 days is similar in 90:10, 80:20, and 70:30 bilayer formulations. There is less increase after 14 days of culture, which the reason might be that the cells reached a plateau stage due to the high initial number per well [45]. The 70:30 bilayer formulation showed the maximum increase in cell proliferation as compared to all other membranes after 3, 7, and 14-day culture. It seems that the pore size, fiber diameter, and mechanical strength of the 70:30 membrane also affected cell attachment and proliferation. These results also support the use of bilayer membranes as biocompatible materials for artificial skin applications.

#### 2.6.2. Scanning Electron Micrographs of MC3T3 Cells Cultured in Electrospun Membranes

**Figure 7:**
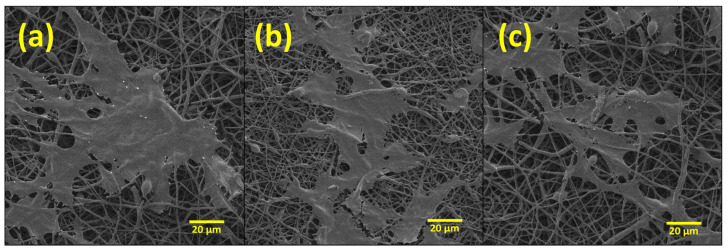
SEM images of PHBV-PLLA bilayer membranes taken after 14 days of culture at different 1k× magnification. (**a**) 90:10, (**b**) 80:20, and (**c**) 70:30 *w*/*w* formulations.

Bilayer membranes of (a) 90:10, (b) 80:20, and (c) 70:30 *w*/*w* formulations of PHBV-PLLA at 1k× were evaluated. SEM images show the membrane surfaces on day 14 after the initial seeding of 50,000 cells. The cells gradually increased from day 1 to 14 and, on day 14, the cells were fixated with glutaraldehyde, washed with 30, 50, 70, 90, and 100 percent alcohol, respectively, and the SEM images of the membranes were taken after drying. Adherent cells on these membranes were observed using the SEM analysis, as the attachment of cells could not be verified with the optical microscope. The cell morphology and the attachment of the cells to the membrane were found to be similar to those reported in the literature [34,35,46], confirming the proliferation of the cells onto the membranes. The confluent layer of MC3T3 cells was attached to the membranes, as shown by SEM (Figure 7). These SEM images validate the biocompatibility of the bilayer membranes by showing cell attachment and growth of the pre-osteoblast MC3T3 cells onto and through the electrospun fibrous mesh. Additionally, it can be concluded that these membranes did not show cytotoxicity to the seeded cells.

#### 2.6.3. Fluorescence Imaging of MC3T3 Cells Stained with DAPI

**Figure 8:**
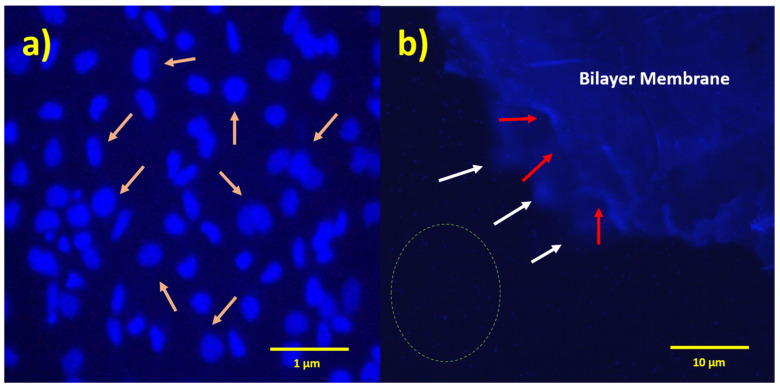
Fluorescent micrographs of PHBV-PLLA bilayer membranes stained with DAPI fluorescent dye. (**a**) Arrows indicate cell nuclei on the membrane, 40× magnification; (**b**) white arrows indicate the edges of the bottom layer of the membrane; red arrows indicate the edges of the upper layer of the membrane; and dotted circle indicates one of the regions in the medium showing the cellular nuclei, 4× magnification.

DAPI staining and fluorescence imaging results also indicate that the membranes are not cytotoxic to the cell, as an increased number of cells on the bilayer membrane are exhibited (Figure 8b). At higher magnification, these layers were filled with cell nuclei, as shown in Figure 8a. It is concluded with this study and Alamar Blue assay that bilayer and monolayer membranes were not cytotoxic and biocompatible with cells.

The fabrication of electrospun membranes for skin tissue applications is gaining interest in biomedical solutions, and the current study aims to highlight this point by providing a proof-of-concept material in the form of a PHBV-PLLA bilayered membrane. All the formulations can show biocompatibility and cellular proliferation in an in vitro mouse skin model (MC3T3 cell). Furthermore, the physical characteristics of these membranes are significant in providing enough mechanical strength as well as support to cell proliferation via its micro/nano-porous structure. Additionally, no significant differences in color or physical appearance of the fibrous mats were seen when placed at room temperature or 40 °C up to 3 months, which shows that these membranes are stable in terms of shelf life.

## 3. Materials and Methods

### 3.1. Materials

Poly (3-hydroxybutyrate-co-3-hydroxyvalerate (PHBV; Goodfellow, Cambridge, UK), methanol (AnalaR Normapur-VWR, Lutterworth, UK), and Dichloromethane (DCM; Sigma Aldrich, London, UK). Poly-l-Lactic Acid (PLLA; Goodfellow, Cambridge, UK). Pre-osteoblast cell line (MC3T3 ATCC^®^ CRL-2593^TM^), alpha minimum essential medium (α-MEM; Thermo Fisher Scientific, Waltham, MA, USA), penicillin/streptomycin (Pen/strep; Sigma Aldrich, Life Sciences, St. Louise, MI, USA), fetal bovine serum (FBS; Sigma Aldrich, USA), T75 culture flask (Corning Biosystem, Flintshire, UK), Trypsin–EDTA (Sigma Aldrich, USA), Alamar Blue solution (Sigma Aldrich, UK), and phosphate-buffered saline (PBS; Gibco, Waltham, MA, USA).

### 3.2. Bilayer Membrane Fabrication

**Figure 9:**
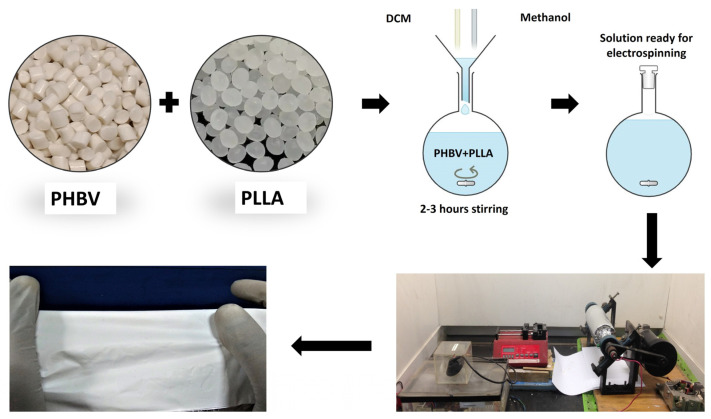
Fabrication of a bilayer electrospun fiber composite membrane.

PHBV was dissolved in a 90:10 DCM methanol ratio followed by the addition and dissolution of Poly-l-Lactic Acid for a composite blend formation. Each bilayer formulation has been named bilayer 90:10, bilayer 80:20, and bilayer 70:30, where 90:10 means weight by weight distribution of 90 against 10 between PHBV and PLLA, respectively. The solution was prepared at room temperature on a mechanical stirrer overnight. On the following day, 3 syringes were filled with 5 mL solutions and were fitted in the multichannel needle holder. The needle gauge was 18 mm in diameter. The syringes were fixed on the (Kent Scientific, Torrington, CT, USA) syringe pump, having a flow rate ranging from 0.35 to 0.45 mL/h. The applied voltage was 15kV with a high-voltage power supply (Genvolt, Bridgnorth, UK). The fibrous scaffold was collected on a rotating drum 16 cm × 6 cm in diameter and wrapped with aluminum foil at a distance of 14 cm from the needle tips with a rotation speed of 300 rpm [34]. An amount of 15 mL of the polymer solution was dispensed to make a fibrous sheet, as described by Bye et al. [46]. Figure 9 shows the schematic fabrication of membranes.

### 3.3. Scanning Electron Microscopy

Scanning Electron Microscopy was performed using TESCAN Vega3 LMU (VEGA TC), using an ETD detector and SE mode after gold coating with a sputter coater from Quorum Technologies. SEM images were acquired using an acceleration voltage of 10 kV with a beam intensity of 4 pA.

### 3.4. Fourier Transform Infrared Spectroscopy (FTIR)

Fourier Transform Infra-Red Spectroscopy (Nicolet 6700, ThermoScientific, Waltham, MA, USA) was performed in Attenuated Total Reflectance (ATR) mode, where each sample was scanned 128 times at a resolution of 8 cm^−1^ in the scan range of 4000–650 cm^−1^.

### 3.5. Mechanical Testing

Mechanical or tensile testing was performed using the Walter + Bai Electrodynamic Fatigue Testing System (LFV-E), having a load of 1.5 KN. The 6 replicates were prepared for each membrane and were cut into a rectangular shape with a width of approximately 5 mm and a length of 45 mm. The thickness of these samples was measured in millimeters with the help of a Vernier caliper with the precision of 0.01 mm. From the slope of the initial linear path of the stress and strain curve, the tensile strength of Young’s modulus was calculated. Strain in percentage and stress in MPa were calculated with the deformation and load values using the following equations [34].

### 3.6. Statistical Analysis of Membranes

With six samples from each of the experiments, all the analysis was conducted at least twice to obtain precise data. From SEM, all the data are shown as the mean. Following Tukey’s post hoc test, a one-way (ANOVA) was performed; *p*-values < 0.05 were considered to be statistically significant. GraphPad Prism 5.0 software was used to analyze all the data.

### 3.7. Analysis of Fiber Thickness and Void Size

Fiber thickness was calculated by selecting at least thirty fibers, each from three frames of images taken using SEM (Version 1.50), using Image-J software (Version 1.54h) [47]. Void and pore sizes in each of the membranes were measured in the same way. The thickness of the electrospun membranes was measured using a microscope, and the apparent density and porosity of the membrane were calculated with the help of the following equations [37].

### 3.8. In Vitro Evaluation

MC3T3 (pre-osteoblastic cell line) cell lines were gifted from National Institute of Biotechnology and Genetic Engineering (NIBGE) Faisalabad, Pakistan. The in vitro biocompatibility test was carried out via cell culture on the membranes using pre-osteoblast cell lines. Cells were expanded in a T75 culture flask (MC3T3) that was prepared, with α-MEM with 100 µg/mL of penicillin/streptomycin and 10% FBS added, in a humidified incubator at a temperature of 37 °C with 5% CO_2_. Fresh media changes were made after 2 to 3 days. The cells were expanded and grown to 90% confluence [44,48]. Afterwards, these cells were detached using trypsin–EDTA. Before seeding, the cells were counted, and 50,000 cells were seeded on each sample in the 24-well plate to check the compatibility of the MC3T3 cells with PHBV-PLLA membranes. Before seeding, the samples were sterilized with 70% ethanol for 2 h. Then, the samples were washed 3 times with PBS at 15-min intervals and the cells were cultured in tissue culture plastic plates without membranes as control.

### 3.9. In Vitro Characterization

#### 3.9.1. Alamar Blue Assay

After 3, 7, and 14 days, absorbance measurements of the Alamar Blue assay were taken to check the biocompatibility of cells with the membranes and the attachment of cells onto the membranes. Based on the metabolic activity of the cells, the Alamar Blue has a redox indicator that changes from an oxidized (blue) form to a reduced (red) form as the substrate is taken up by the cells. Cell-seeded samples were carefully washed with PBS and 0.5 mL of 1mM Alamar Blue solution was added. After that, it was incubated for 3 to 4 h at 37 °C. An absorbance plate reader (PR4100 Absorbance Microplate Reader BIO-RAD, Hemel Hempstead, UK) was used to measure the absorbance at 570 nm. Samples were fixed for DAPI staining and SEM analysis after the results were taken from the plate reader. To prepare the sample for SEM, the membranes were dehydrated and washed with the different dilutions (30, 50, 70, 90, and 100%) of ethanol and then dried.

#### 3.9.2. Fluorescence Microscopy

Fluorescence microscopy was performed using an 89404-464 VWR microscope having 240 V with a frequency of 50–60 Hz and a Halogen lamp of 6 V and 30 W. The live cells were stained with DAPI and fluorescence images were taken after excitation with UV light with a 460 nm filter at 10× magnification.

## 4. Conclusions

Biocompatible PHBV-PLLA bilayer membranes containing different concentrations (70:30, 80:20, and 90:10 *w*/*w*) of each polymer in the blend were developed through electrospinning. SEM analysis shows the fibrous non-woven structure of the electrospun matrix with the increase in pore diameter and fiber thickness with higher PLLA content in the blends. The pore size of the 90:10 bilayer formulation was measured to be 1.8 µm and the fiber diameter 0.6 µm, while the pore size of the 70:30 bilayer was 2.1 µm and fiber diameter was 0.7 µm, respectively. The 90:10 bilayer formulation showed significantly higher tensile strength compared to the PHBV and PLLA monolayers, i.e., 7.940, 4.224, and 2.450 MPa, respectively. In addition, there was a significant increase in the tensile strength for higher concentrations of PHBV in the blended bilayer structures. In vitro biocompatibility proved that all three ratios of the bilayer membrane were highly biocompatible. Cell proliferation increased after culturing on the membranes for 3 and 7 days, with no cytotoxicity. The highest cell proliferation was observed in the 70:30 bilayer membrane, with the highest pore diameter around 2 µm. The tensile strength of bilayers with smaller pore sizes was highest, i.e., 172 MPa, but the cell proliferation rate was lowest (Figure 6), while the mechanical strength of bilayers with larger pore sizes had lower tensile strength but significantly higher cell proliferation. This research can be useful in developing biomedical materials for skin tissue scaffolds. Future work includes the seeding and culture of keratinocytes and fibroblasts.

## Figures and Tables

**Table 1:** Morphological properties of the PHBV and PHBV-PLLA bilayer membranes.

Membranes	Bulk Density	Thickness	Porosity
PHBV	1.25 g	70 µm	65%
PHBV-PLLA bilayer	1.26 g	180 µm	67–70%

**Table 2:** Mechanical properties of PHBV-PLLA membranes were calculated from stress–strain curves. (* Young Modulus rating of submandibular skin by Griffin et al. [41]).

Membrane Type	Young Modulus MPa	Ultimate Tensile Stress MPa	Elongation at Break %
Submandibular skin	1.28 MPa *	-	-
PLLA	45.65	2.450	40.05
PHBV	119.7	4.224	28.41
Bilayer 90:10	172.0	7.940	44.45
Bilayer 80:20	144.1	6.355	37.63
Bilayer 70:30	128.2	5.470	33.95

## Data Availability

Data will be made on available request.
